# Deep-learning based 3D reconstruction of lower limb bones from biplanar radiographs for preoperative osteotomy planning

**DOI:** 10.1007/s11548-024-03110-5

**Published:** 2024-04-04

**Authors:** Tabitha Arn Roth, Moritz Jokeit, Reto Sutter, Lazaros Vlachopoulos, Sandro F. Fucentese, Fabio Carrillo, Jess G. Snedeker, Hooman Esfandiari, Philipp Fürnstahl

**Affiliations:** 1 Institute for Biomechanics, ETH Zurich, Zurich, Switzerland; 2 Research in Orthopedic Computer Science (ROCS), University Hospital Balgrist, University of Zurich, Balgrist Campus, Lengghalde 5, 8008 Zurich, Switzerland; 3 Department of Radiology, Balgrist University Hospital, University of Zurich, Forchstrasse 340, 8008 Zurich, Switzerland; 4 Department of Orthopedics, Balgrist University Hospital, University of Zurich, Forchstrasse 340, 8008 Zurich, Switzerland

**Keywords:** 2D–3D reconstruction, 3D preoperative planning, High tibial osteotomy, EOS imaging system, Deep learning, Weight-bearing

## Abstract

**Purpose:**

Three-dimensional (3D) preoperative planning has become the gold standard for orthopedic surgeries, primarily relying on CT-reconstructed 3D models. However, in contrast to standing radiographs, a CT scan is not part of the standard protocol but is usually acquired for preoperative planning purposes only. Additionally, it is costly, exposes the patients to high doses of radiation and is acquired in a non-weight-bearing position.

**Methods:**

In this study, we develop a deep-learning based pipeline to facilitate 3D preoperative planning for high tibial osteotomies, based on 3D models reconstructed from low-dose biplanar standing EOS radiographs. Using digitally reconstructed radiographs, we train networks to localize the clinically required landmarks, separate the two legs in the sagittal radiograph and finally reconstruct the 3D bone model. Finally, we evaluate the accuracy of the reconstructed 3D models for the particular application case of preoperative planning, with the aim of eliminating the need for a CT scan in specific cases, such as high tibial osteotomies.

**Results:**

The mean Dice coefficients for the tibial reconstructions were 0.92 and 0.89 for the right and left tibia, respectively. The reconstructed models were successfully used for clinical-grade preoperative planning in a real patient series of 52 cases. The mean differences to ground truth values for mechanical axis and tibial slope were 0.52° and 4.33°, respectively.

**Conclusions:**

We contribute a novel framework for the 2D–3D reconstruction of bone models from biplanar standing EOS radiographs and successfully use them in automated clinical-grade preoperative planning of high tibial osteotomies. However, achieving precise reconstruction and automated measurement of tibial slope remains a significant challenge.

## Introduction

Over the past years, preoperative planning in orthopedic surgery has undergone notable transformations, driven by technological advancements and the introduction of novel tools such as patient-specific instruments (PSI) [6]. Three-dimensional (3D) planning has become an integral part of surgical procedures [7], albeit often requiring laborious and costly processes. Based on CT-reconstructed 3D models, the surgeries are meticulously planned by biomedical engineers. They define osteotomy cuts, calculate correction angles, and determine the position of implants (e.g. fixation plates) such that the targeted correction is achieved as accurately as possible. They do this while considering all clinically necessary criteria and constraints for the placement of osteotomy cuts and implants. In cases with multiplanar deformities, this process becomes particularly complex. In the end, the planning is optimized through several rounds of discussions with the treating surgeons. A more detailed description of the preoperative planning process can be found in our previous publications [7, 10].

Currently, 3D planning requires the acquisition of a CT to reconstruct 3D models of the patient anatomy, which serve as the basis to simulate and define each step of the surgery. However, for many surgeons, the 3D planning alone is not deemed mandatory due to radiation exposure concerns. Furthermore, the CT acquisition and the consequent 3D planning is done in the supine position and therefore does not allow the deformity assessment in the weight-bearing state. As a consequence, most surgeons resort to the traditional 2D approach using standing radiographs. An imperative advantage of utilizing radiographs for surgery planning purposes lies in their ability to capture the lower limb in a standing position, providing valuable information for biomechanical assessments [10]. Compared to CT scans, standing X-rays offer a representation of the weight-bearing situation, which is relevant for the development of osteoarthritis (OA). The EOS imaging system (EOS imaging system, EOS, Paris, France) has therefore become increasingly widespread used in over 400 locations worldwide [8]. Although the system comes at high cost, it features the acquisition of calibrated biplanar (90 degrees) standing radiographs at an ultra-low dose, which is 50% lower than a standard X-ray [4].

Reconstructing 3D models based on standing radiographs combines the benefits of both worlds: It facilitates 3D planning that is based on imaging data acquired in a weight-bearing position with a reduced radiation dose. This exhibits notable potential in enhancing the accuracy of preoperative planning [17], particularly for procedures involving anatomical structures that are influenced by posture, such as the lower limb or potentially the spine. In this study, we investigated the feasibility of performing 3D planning for high tibial osteotomy (HTO) surgery without the need of a CT scan by reconstructing a 3D surface model of the proximal tibia solely from biplanar radiographs acquired with the EOS system, aiming to enhance efficiency, reduce healthcare costs, and concurrently minimize radiation exposure for the patient.

2D-3D reconstruction from X-ray imagery has been traditionally achieved by statistical shape models (SSM), which are anatomical atlases created based on a large patient database. In this approach, the parameters of an SSM are optimized to match a 2D projection of the SSM and the contour of the bone seen in the radiograph. The matching process, however, is sensitive to initialization [12, 15, 21]. However, the major drawback of SSM is its inability of representing patient-specific pathologies. Recent studies have proposed deep-learning-based approaches for patient-specific reconstruction of anatomies based on X-ray data for different applications such as the spine [2, 3, 9] or the knee joint [11]. Kasten employed an end-to-end trained CNN to reconstruct the proximal tibia, distal femur, proximal fibula, and patella from conventional radiographs and achieved accurate 3D reconstructions with Dice scores between 0.85 and 0.95. However, they have reconstructed the 3D models from radiographs including only one knee joint. To calculate the required planning parameters (e.g., the mechanical axis angle, defined by hip, knee and ankle joint centers), surgeons often require a full standing leg radiograph, which always includes both legs from hip to ankle joints. As a consequence of this imaging setup, the two legs are at least partially superimposed in the sagittal image, making the 3D reconstruction task more challenging.

To address the aforementioned challenges, we have designed a deep-learning based pipeline to reconstruct 3D surface models of the proximal tibial from biplanar standing EOS radiographs for the purpose of 3D preoperative planning. In a first step, we localize several clinically required landmarks in the biplanar radiographs. Thereafter, two separate sagittal images (each containing one leg) are generated from the original sagittal radiograph. The frontal and the separated sagittal image are then used to reconstruct the 3D surface model of the proximal tibia, which is finally used as an input to our automated preoperative planning pipeline for high tibial osteotomy (HTO) surgeries. For evaluation, we compare the solutions to the ground truth solutions generated based on CT-reconstructed 3D models using a series of 52 patients. In summary, (1) our novel separation network improves the 3D-reconstruction of anatomical regions that overlap in X-ray data and (2) we clinically evaluate the usability of the 2D-3D reconstructed models in a fully automated preoperative planning framework. The entire pipeline is designed for using radiographs from the EOS imaging system, a globally widespread, pre-calibrated, low-dose biplanar imaging system.

## Methods

**Fig. 1:**
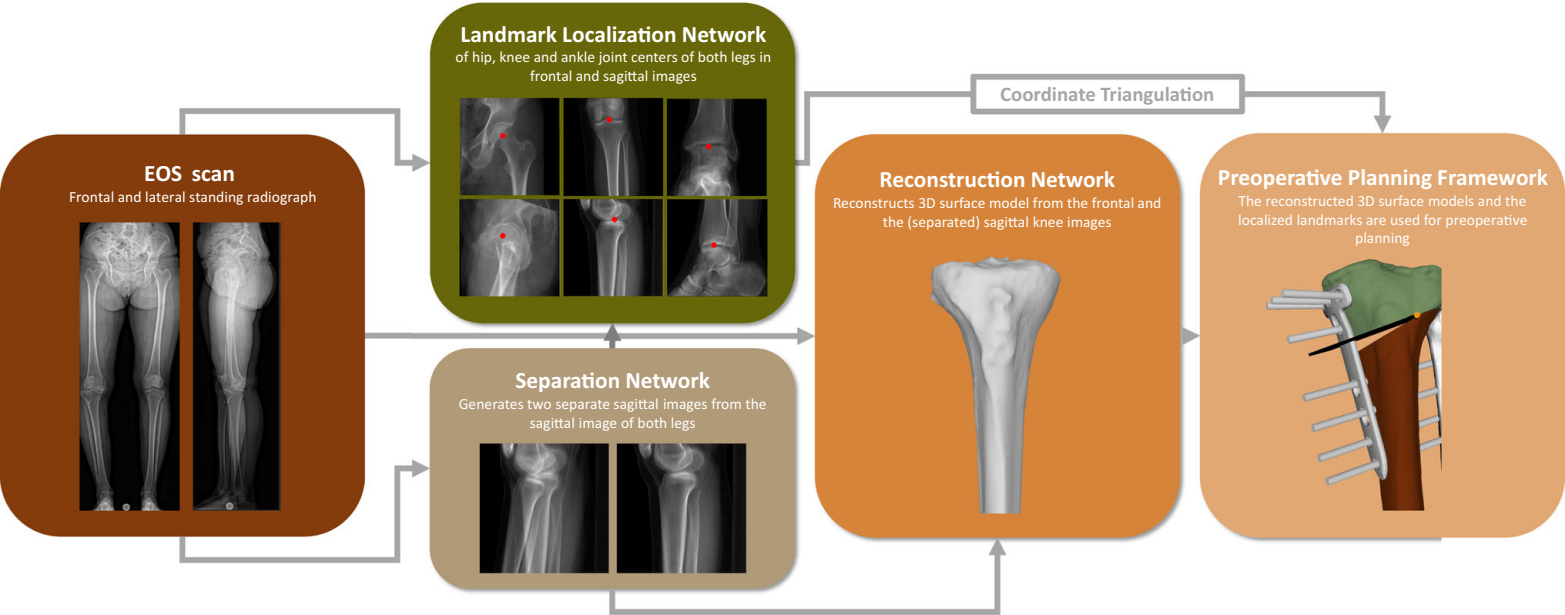
The proposed pipeline. A biplanar EOS scan serves as input. The separation network separates the two legs into two separate sagittal images. The frontal and the separated sagittal image are used both for the localization of the landmarks required for orthopaedic measurements and for reconstruction. The localized 2D landmarks are triangulated to obtain 3D landmarks. Together with the reconstructed 3D surface models, they are the input to the preoperative planning framework, which generates ready-to-use preoperative planning solutions

Our proposed pipeline for 2D–3D reconstruction is depicted in Fig. 1. A biplanar EOS scan is used as input. A separation network is first designed to generate separated sagittal images of both legs each containing the projection of a single leg based on one original sagittal EOS image. Additionally, a localization network determines the coordinates of the joint centers which are required for orthopedic measurements and preoperative planning purposes. The frontal EOS image along with the separated sagittal EOS images are then used as the input to a reconstruction network that is tasked with producing the required 3D models. Finally, the triangulated 3D joint coordinates and the reconstructed 3D model of the knee serve as input for a preoperative planning framework, which automatically generates the desired preoperative planning solution. This includes the positioning of the osteotomy axis, the calculation of the opening angle, as well as the placement of the fixation plate and screws (Fig. 6C), all while adhering to clinically necessary constraints and rules.

### Dataset generation

The study was approved by the local ethics committee and informed consent was obtained from all patients (Zurich Cantonal Ethics Commission, KEK 2018-02242). To train the AI networks involved in our pipeline, we utilized a dataset of 175 HTO patients who underwent CT scans of both legs (Philips Brilliance 64, Philips Healthcare, Best, The Netherlands, or Somatom Definition AS Siemens Healthcare, Erlangen, Germany). Patients with only a unilateral CT scan of the pathological leg were excluded. For the evaluation in section "Preoperative planning accuracy" we have used a different set of patients, which was the same set as in our previous publication [16].

The CT scans were obtained following the MyOsteotomy protocol, which involved separate scans for the hip, knee, and ankle joints (whilst skipping the bone shafts) to minimize radiation exposure. Prior to our study, these CT scans were segmented using commercial segmentation software (Mimics Medical 19.0, Materialise NV, Leuven, Belgium) and the hip, knee and ankle joint centers (HJC, KJC, AJC) were manually annotated. The HJC was defined as the center of a sphere fitted to the femoral head while the KJC was located between the two tibial eminences. The AJC was determined by calculating the center of all points of the distal tibial and fibular articular surfaces (see [7] for details).

**Fig. 2:**
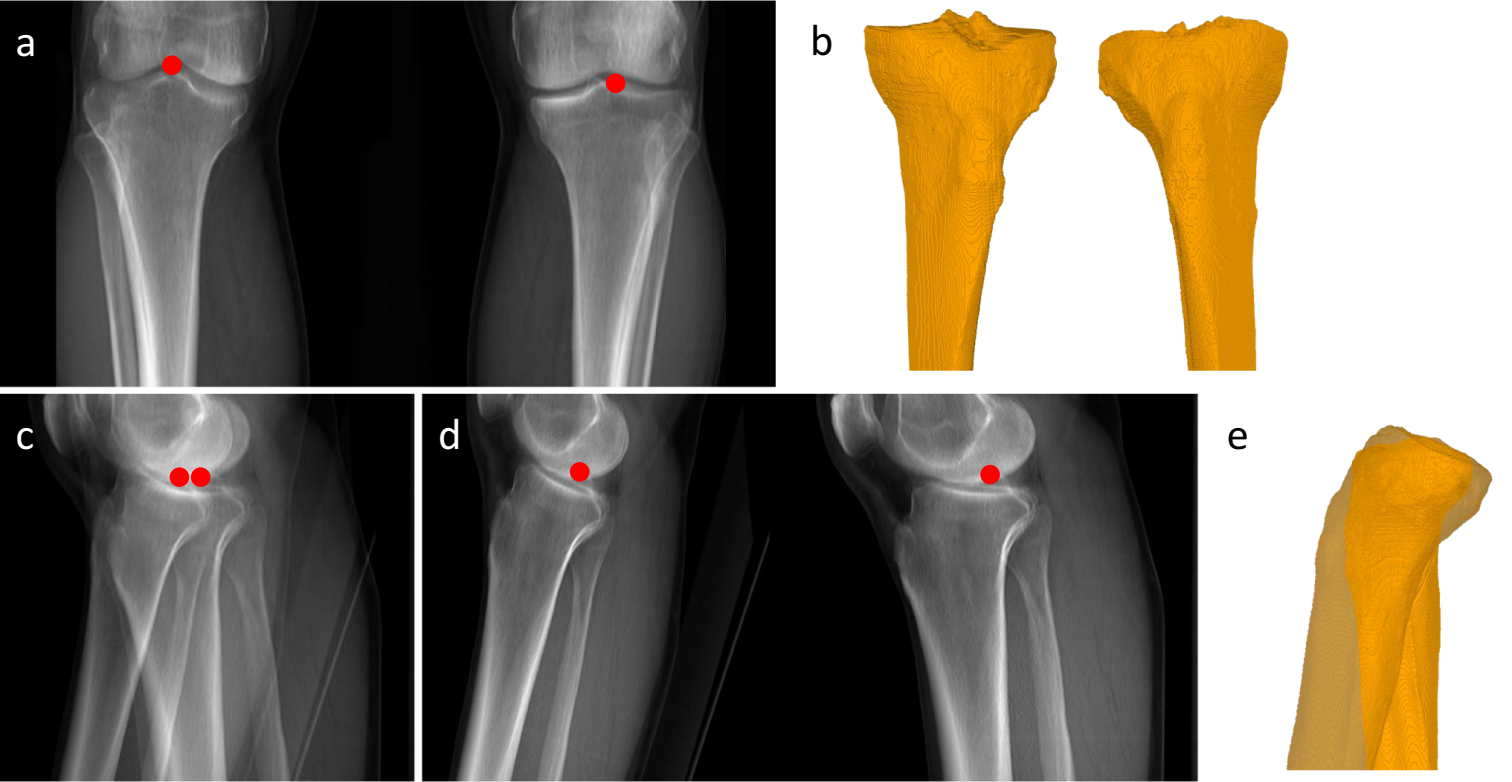
DRR-generated images for one patient. **a** Frontal image, **b** ground truth 3D model frontal view, **c** sagittal image of both legs, **d** sagittal images of single legs, **e** ground truth 3D model sagittal view

To facilitate a reliable comparison between the 3D reconstructions generated by our neural network and the ground truth, in this study, we have trained and tested our networks using digitally reconstructed radiographs (DRR). To this end, we have used segmented CT scans of 175 patients and developed a DRR generation method specifically for the geometry of the EOS imaging system, which utilizes a unique biplanar imaging geometry with a moving fan beam emitter. We have described the EOS imaging system in our previous publication [16]. Using a CT scan as the input, we generated a frontal and sagittal image for each patient in our dataset, ${I}_{fron}$ and ${I}_{sag}$. Besides the normal sagittal image of both legs, we additionally generated sagittal images containing only the left or the right leg, respectively, as the targets for our separation network (${I}_{sag}^{L}$ and ${I}_{sag}^{R}$). At the same time, the 3D landmarks annotated in the CT are utilized and projected to the frontal and sagittal image planes to obtain the ground truth 2D landmark coordinates required for the landmark localization network (Fig. 2).

In clinical practice, when acquiring an EOS image, patients are instructed to position their right foot slightly in front of their left foot to enhance the distinguishability of the two legs in the sagittal image. However, despite this positioning, a certain degree of superimposition remains. To mimic this positioning in our DRR generation process, we randomly applied shifts (40 to 60 mm) and rotations (10° to 20° around the center of the image) in the sagittal plane to the CT scan of the right leg before generating the image. To augment our dataset, this process was repeated three times for each patient, resulting in a final dataset consisting of 525 biplanar image pairs, each comprising a frontal and a sagittal DRR, along with their corresponding ground truth 3D bone models. The subsequent splitting of data into train and test sets was on patient level to prevent the mixing of patients between the sets. The same split was used for all three networks.

### Leg separation network

**Fig. 3:**
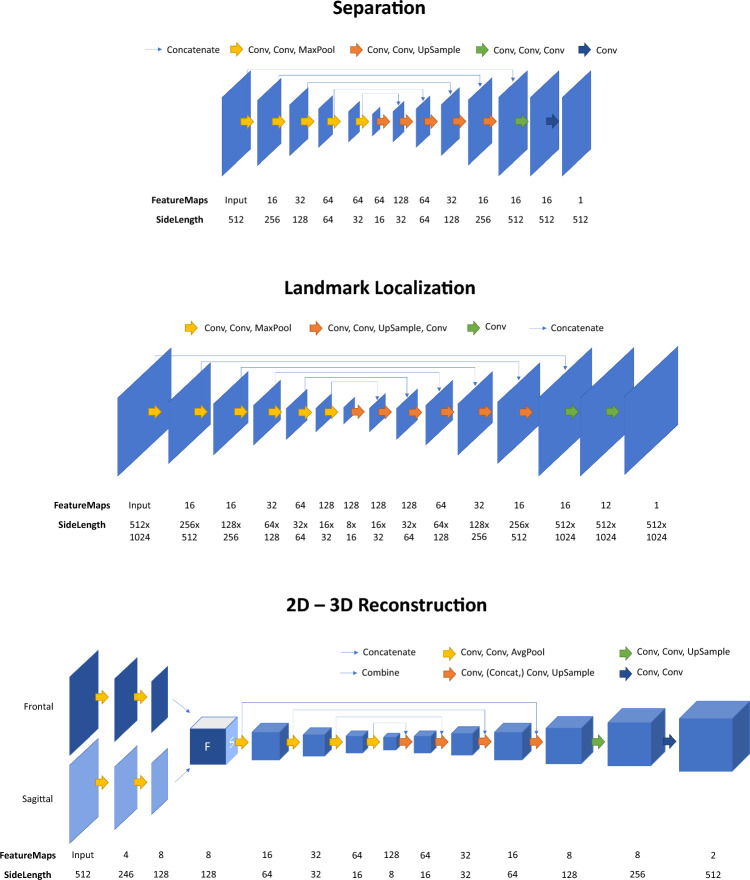
Model architectures of the separation, landmark localization and 2D – 3D reconstruction networks

We designed a dedicated network capable of separating the two legs in the sagittal radiograph, allowing us to obtain individual images for each leg. To this end, we employed a CNN, which was trained using the original DRR as the input and the two separated DRRs as the targets. The CNN architecture consisted of one encoding path and two separate decoding paths, each corresponding to one output image. Model architectures are shown in Fig. 3, details can be found in Table 1.

**Table 1:** Network parameters for the separation, the landmark localization and the 3D reconstruction networks

	Separation	Landmark localization	3D reconstruction
Convolution Kernel size	3	3	3
Pooling size	2	2	2
Activation function	ReLu	ReLu	ReLu
Padding	‘same’	‘same’	‘same’
Weight initialization	‘he_normal’	‘he_normal’	‘he_normal’
Final layer activation function	‘sigmoid’	‘sigmoid’	‘softmax’
Learning rate	0.001	0.0001	0.00005
Optimizer	Adam	Adam	Adam
Loss function	MSE + GCOR	Binary Crossentropy	Dice + Crossentropy
Epochs	20	20	30
Batch size	16	1	1
Number of samples	525	525	525
Train (train/val)/test split	90 (80/20)/10	90 (80/20)/10	90 (80/20)/10

**Fig. 4:**
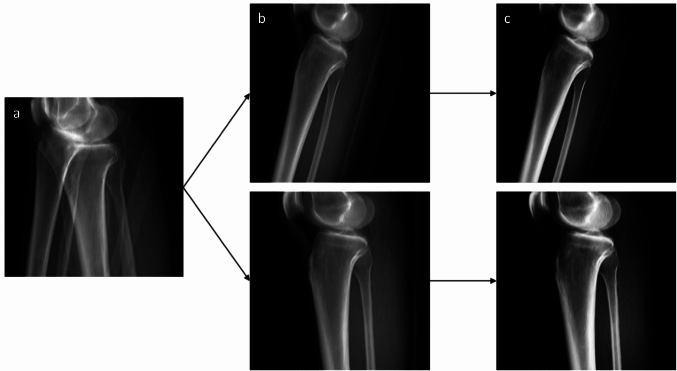
**a** Input to the separation network: sagittal DRR of both legs. **b** Sagittal DRRs of right (top) and left (bottom) leg. **c** Contrast enhanced sagittal DRRs, used as targets for the separation network

When directly using ${I}_{sag}^{L}$ and ${I}_{sag}^{R}$ as the target images, our network encountered difficulties in discerning between the contralateral leg, which needed to be removed from the image, and the surrounding soft tissue, that should be preserved. To address this issue, we applied a contrast enhancing transformation to the pixel values $x$ of ${I}_{sag}^{L}$ and ${I}_{sag}^{R}$ which is represented in Eq. (1), facilitating the isolation of the bone structures from the surrounding soft tissue (Fig. 4). The parameters of Eq. (1) were determined empirically.

1 $$f(x)=\frac{1}{1+ {e}^{-\frac{x-0.45}{0.12}}}$$

The loss function ${\text{L}}(y,\widehat{y})=\frac{1}{2}\left(\frac{1}{{\text{N}}}\sum_{{\text{i}}=1}^{{\text{N}}}{({{\text{y}}}_{{\text{i}}-}{\widehat{{\text{y}}}}_{{\text{i}}})}^{2}+ {{\text{L}}}_{{\text{GC}}}(y,\widehat{y})\right)$ comprised a combination of the mean squared error (MSE) loss and gradient correlation (Eq. 2), where the ground truth and the predictions are represented by *y* and $\widehat{y}$, respectively.

The gradient correlation metric is based on the horizontal and the vertical image gradients and is used to improve the clarity and sharpness of the leg outlines in the separated radiographs [5]. ${\mu }_{\lambda }$ and ${\mu }_{\kappa }$ are the mean pixel values of the horizontal and vertical gradient images, respectively.

2 $${L}_{GC}\left(y, \widehat{y}\right)=\frac{1}{2} {(NCC}_{x}(y, \widehat{y})+ {NCC}_{y}(y, \widehat{y}))$$

3 $${NCC}_{x}\left(y, \widehat{y}\right)= \frac{{\sum }_{i}({\lambda }_{1}\left(i\right)- {\mu }_{{\lambda }_{1}})({\lambda }_{2}\left(i\right)- {\mu }_{{\lambda }_{2}})}{\sqrt{{\sum }_{i}({{\lambda }_{1}\left(i\right)- {\mu }_{{\lambda }_{1}})}^{2}}* \sqrt{{\sum }_{i}({{\lambda }_{2}\left(i\right)- {\mu }_{{\lambda }_{2}})}^{2}}}$$

4 $${NCC}_{y}\left(y, \widehat{y}\right)= \frac{{\sum }_{i}({\kappa }_{1}\left(i\right)- {\mu }_{{\kappa }_{1}})({\kappa }_{2}\left(i\right)- {\mu }_{{\kappa }_{2}})}{\sqrt{{\sum }_{i}({{\kappa }_{1}\left(i\right)- {\mu }_{{\kappa }_{1}})}^{2}}* \sqrt{{\sum }_{i}({{\kappa }_{2}\left(i\right)- {\mu }_{{\kappa }_{2}})}^{2}}}$$

### Landmark localization network

Another CNN was designed to localize the three joint center landmarks of hip, knee and ankle joints (${H}_{C}$, ${K}_{C}$, ${A}_{C}$) in ${I}_{fron}$ as well as ${I}_{sag}^{L}$ and ${I}_{sag}^{R}$. To generate the training data, 3D landmarks of the ground truth CT data were projected as 2D landmarks onto the DRR-generated images. These coordinates were used to generate target heatmaps, containing a Gaussian distribution of values between 0 and 1 around the ground truth joint center location. The model architecture is depicted in Fig. 3, additional raining and network details can be found in Table 1. The binary crossentropy loss is defined as

5 $$L\left(y, \widehat{y}\right)=-\frac{1}{N}\sum_{i=1}^{n}{y}_{i}\mathit{log}\left({\widehat{y}}_{i}\right)+\left(1-{y}_{i}\right)log(1-{\widehat{y}}_{i})$$

### 3D reconstruction network

Similar to [11], we designed a U-net for 2D–3D reconstruction of 3D bone models of the proximal tibia from the frontal as well as the separated sagittal DRR. As the target, we used the segmentation label maps of the CTs from which the DRRs were generated. For the first two levels of the CNN, the frontal and sagittal 2D images are processed in two separate network branches. After two levels, 2D feature maps (size 128 × 128) were replicated 128 times over the third dimension to obtain arrays of size 128 × 128 × 128. The arrays were then fused into a two-channel representation and subsequently averaged per voxel, resulting in 3D feature maps of size 128 × 128 × 128. The rest of the encoding as well as the decoding path was performed in 3D. Skip connections were used on all levels except the two top layers. The model architecture is shown in Fig. 3, training details are summarized in Table 1. As a loss function we used the sum of the Dice and the crossentropy loss:

6 $$L\left(y, \widehat{y}\right)=\sum_{i=1}^{n}1-\frac{2\sum {y}_{i}*{\widehat{y}}_{i}}{\sum {y}_{i}+ \sum {\widehat{y}}_{i}}-{y}_{i}log({\widehat{y}}_{i})$$

The final 3D bone models were obtained by applying a Marching Cubes algorithm [13] to the 3D binary output arrays.

### Surgery planning

**Fig. 5:**
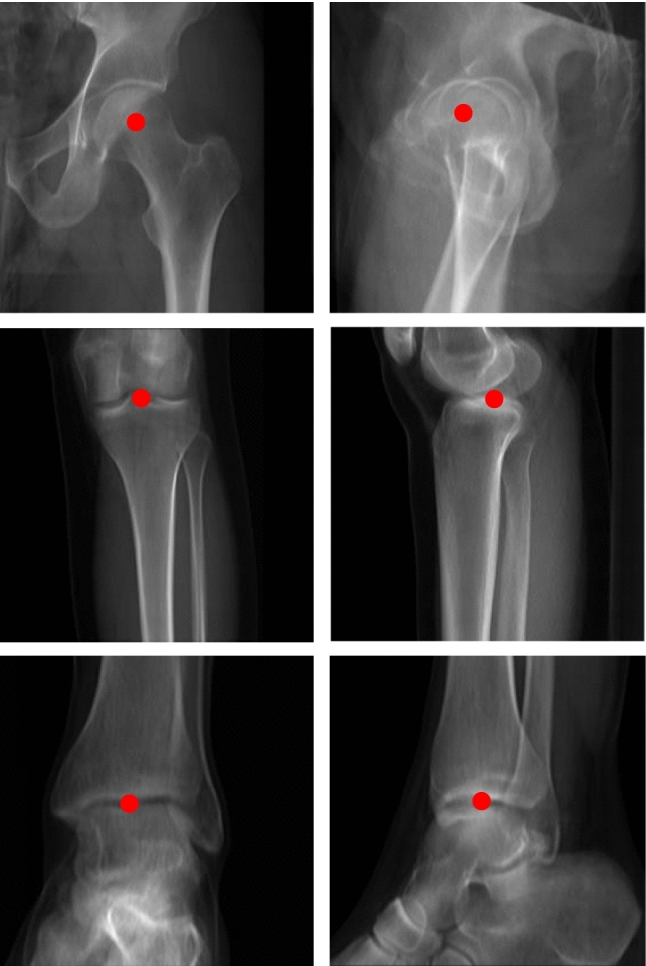
An example for the localized landmarks of a left leg in the frontal and sagittal planes

Finally, we have integrated our previously validated preoperative planning framework [16] in the current pipeline. The framework is based on a genetic algorithm for multi-objective optimization (MOO) and takes a 3D model of the proximal tibia, the 3D landmark coordinates for hip, knee and ankle joint centers ${H}_{C}^{3D}$, ${K}_{C}^{3D}$ and ${A}_{C}^{3D}$ (Fig. 5) as well as target values ${\Phi }_{MA}$ and ${\Phi }_{TS}$ for the two anatomical deformity measurements (mechanical axis (MA) and the tibial slope (TS)) as input.

**Fig. 6:**
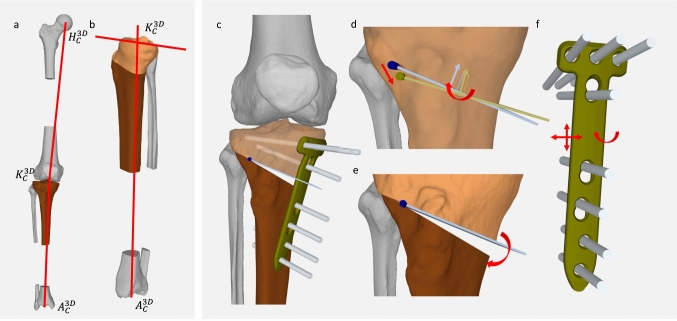
Anatomical deformity measurements and evaluation of surgery planning. **a** The MA is defined by the hip, knee and ankle joint centers. **b** The TS is defined by the tibial mechanical axis (${{\text{K}}}_{{\text{C}}}$ to ${{\text{A}}}_{{\text{C}}}$) and the tibial slope plane. **c** Surgery planning includes the placements of osteotomy axis, osteotomy plane and fixation plate as well as the calculation of the correction angle. The planning solutions were evaluated based on **d** the Euclidean distance between the ground truth (green) and predicted (blue) osteotomy axes as well as the orientation of the osteotomy plane normal, **e** the difference in correction angle and **f** the translational and rotational differences of the fixation plate position

Based on the joint center landmarks, the framework calculates the patient's deformity measurements (Fig. 6A, B). The MA is defined as the angle between the two lines connecting the ${H}_{C}^{3D}$, ${K}_{C}^{3D}$ and ${A}_{C}^{3D}$, projected to the frontal plane. A plane fitting algorithm is used to find the articular surface plane of the proximal tibia, and the angle between its normal and the line connecting ${K}_{C}^{3D}$ and ${A}_{C}^{3D}$, projected to the sagittal plane, is defined as the TS.

The MOO framework subsequently optimizes a set of twelve osteotomy parameters to find the ideal solution. The twelve osteotomy parameters comprise values for the position and orientation of the osteotomy axis (4), the osteotomy angle (1), the fixation plate position (3) and orientation (3) as well as the inclination angle of the osteotomy plane (1). The quality of a solution is assessed by three fitness functions, measuring (1) the deviation to the target MA, (2) the deviation to the target TS and (3) the mean distance between the fixation plate and the bone. The optimization is guided by non-linear constraints which are formulated based on clinical requirements regarding the positioning of the axis, cutting plane and fixation plate.

### Evaluation

The precision of the localized landmarks was evaluated by calculating the Euclidean distance to the ground truth landmarks. The performance of the separation network was assessed by performing an ablation study which compared the performance of the reconstruction network with and without prior separation.

The accuracy assessment of the entire deep-learning based 3D reconstruction pipeline was assessed by two metrics. First, Dice scores were computed between the predicted and ground truth segmentation label maps. Secondly, the mean Euclidean distance between the predicted and ground truth meshes was calculated by averaging the distances between each vertex in the predicted and its closest point in the ground truth model.

Finally, the feasibility for using the reconstructed 3D models for preoperative planning was evaluated by comparing the results to the solutions using ground truth 3D bone models as depicted in Fig. 6C–F. The same patient series was used as in the previous publication, except for one patient who was excluded due to a pre-existing implant.

## Results

### 3D reconstruction

**Fig. 7:**
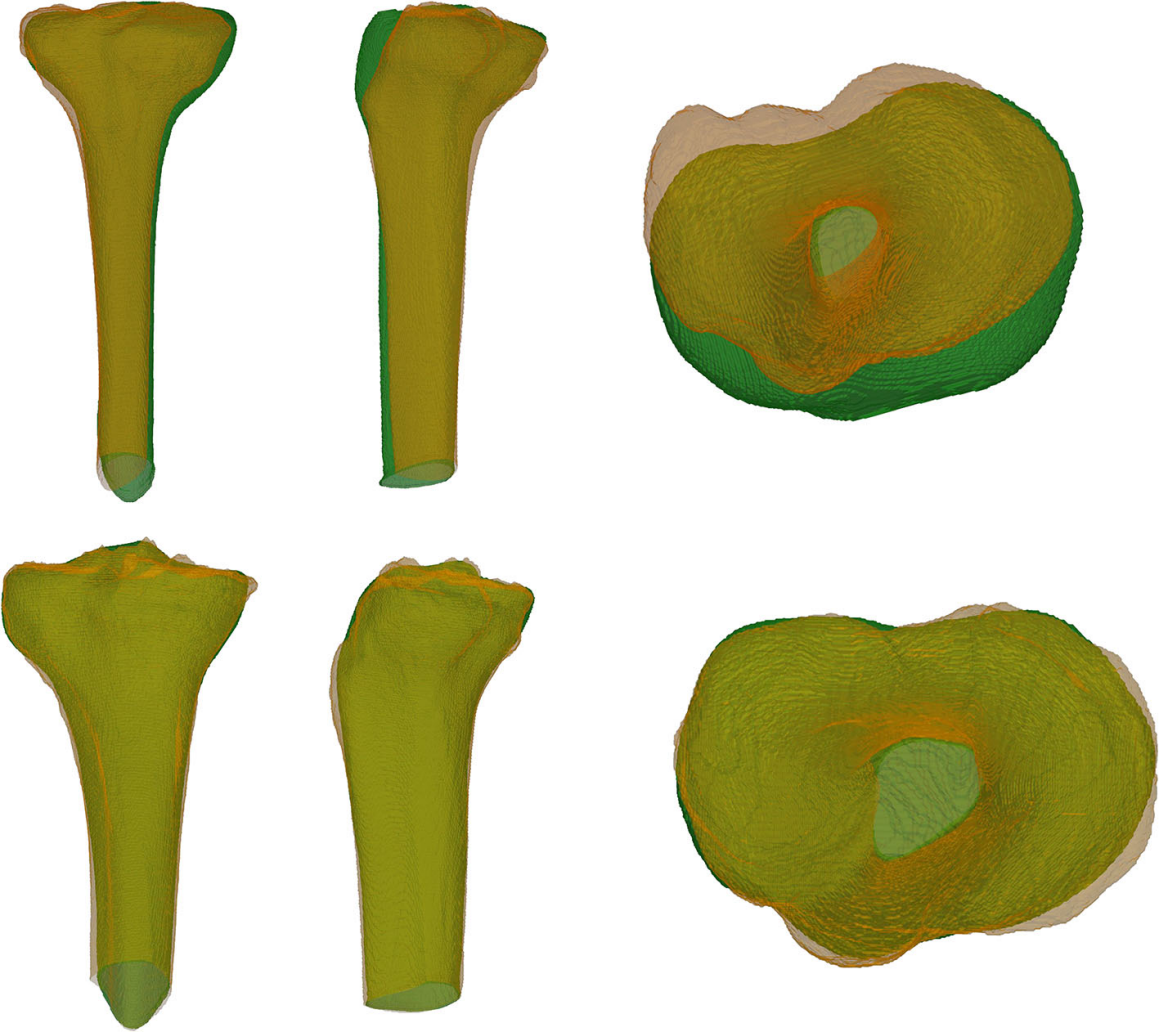
The two test set examples with the highest and lowest Dice scores. The ground truth models are displayed in orange, the reconstructed models in green. The Dice coefficient of the shown examples are 0.83 (top), and 0.96 (bottom)

The Dice coefficients for the reconstructed proximal tibiae in the test set were 0.92 ± 0.02 and 0.89 ± 0.06 for the right and left sides, respectively. The mean Euclidean distances between closest points were 1.21 ± 0.38 mm and 1.63 ± 0.74 mm for the right and left side. Two examples are presented in Fig. 7.

### Landmark localization

Mean localization error in the frontal plane for hip, knee and ankle joints were 1.78 ± 1.19 mm, 1.64 ± 0.97 mm and 1.64 ± 0.91 mm, respectively. In the sagittal plane, the same mean errors were 3.72 ± 2.45 mm, 2.71 ± 2.41 mm and 1.84 ± 1.16 mm.

This resulted in a mean 3D Euclidean distance to the ground truth landmarks of 2.87 ± 1.37 mm, 3.63 ± 2.35 mm and 2.87 ± 1.29 mm, leading to a mean difference in the measured MA of 0.52° ± 0.47° between the ground truth and the reconstructed models. The mean absolute measured difference for the TS was 4.33° ± 3.92°.

### Separation

**Fig. 8:**
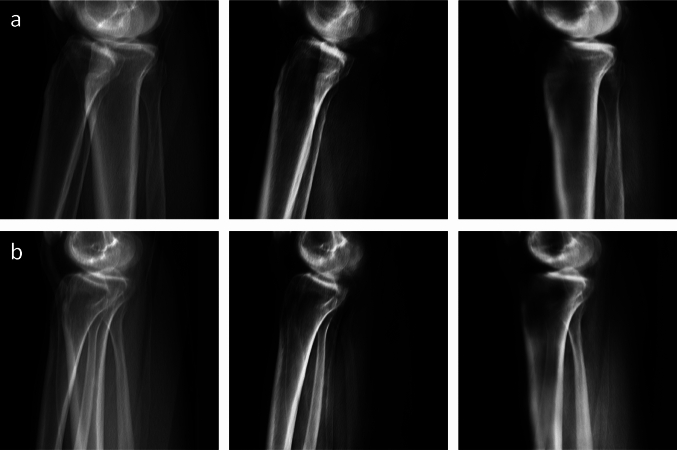
Two examples of our separation results. The input image is shown in the first column, the second and third column show the separated images (right and left leg, respectively)

An ablation study was performed and resulted in a Dice coefficient of 0.90 ± 0.07 and 0.85 ± 0.03 for the right and the left side, respectively. Therefore, the separation network could improve the reconstruction performance by 2.2% and 4.7%, respectively. Two examples are shown in Fig. 8.

### Preoperative planning accuracy

The trained networks were applied to the series of 52 patients, which was used for validating our preoperative planning pipeline [16]. Our algorithm found clinically feasible solution for all patients. We compared the preoperative planning solutions that were obtained based on the 2D–3D reconstructed models with the previously generated ground truth solutions, which were based on the CT-reconstructed models (Fig. 6C–F).

#### Fitness values

We assessed the fitness of a given solution by calculating (1) the deviation from the target MA, (2) the deviation from the target TS and (3) the mean difference between the fixation plate and the bone surface. The mean absolute difference between the target MA and the achieved MA is 0.06° ± 0.11° and 0.11° ± 0.33° for the ground truth and the reconstruction solutions, respectively. The mean absolute difference between the target TS and the achieved TS is 0.04° ± 0.13° and 0.80° ± 2.13°. The mean difference between the bone and the fixation plate was 2.28 ± 1.48 mm for the ground truth solutions and 2.34 ± 1.55 mm for the reconstruction solutions.

#### Osteotomy planning

The mean absolute Euclidean difference for the position of the osteotomy axis in the frontal plane was 2.93 ± 1.90 mm, resulting from a difference of 2.27 ± 1.88 mm in the medial–lateral direction and 1.52 ± 1.11 mm in the superior-inferior direction (Fig. 6D).

The normal vector of the osteotomy plane was projected to the frontal and to the sagittal plane to assess the deviation to the normal vector in the ground truth solution. The measured differences were 2.16° ± 1.73° and 8.32° ± 6.45° for the frontal and sagittal plane, respectively (Fig. 6D).

The mean difference for the correction angle was 1.25° ± 1.14° (Fig. 6E).

The mean absolute 3D difference of the fixation plate was 7.06 ± 7.50 mm in 3D. The 2D error in x, y and z direction was 2.54 ± 2.84 mm, 2.41 ± 1.97 mm and 5.34 ± 7.32 mm, respectively. The mean absolute angular difference of the fixation plate position was 4.95° ± 5.29°, 3.1° ± 2.36° and 4.30° ± 4.04° (Fig. 6F).

## Discussion

2D-3D reconstruction is a highly active research topic in general computer vision and more recently in medical imaging research. CT scans are not only expensive and expose the patients to a high ionizing radiation dose but are also acquired in a non-weight-bearing position. Therefore, multiple research groups have tried to reconstruct accurate 3D bone models from standing 2D radiographs. Various approaches have been tried for spine [2, 9] and lower limb reconstruction [1, 11].

In our study, the specific aim was to investigate whether 2D-3D reconstructed models are sufficiently accurate to be used for clinical-grade preoperative planning [16]. The EOS imaging system is an emerging imaging technology, providing low-dose biplanar standing radiographs at a fixed angle of 90°. Hence, this imaging modality was used as the basis for our 2D–3D reconstruction task. To this end, we used a CNN algorithm to perform the reconstruction task. As for the training data, we created EOS DRRs from a large dataset of patient CT scans. The 3D models were successfully used for preoperative planning and yielded similar planning solutions to the ground truth models. With the separation network, we additionally contributed to the processing of long-leg standing radiographs by addressing the issue of superimposition in the sagittal images. In the separated image of the left leg, we often observed blurry contours, particularly in the area of the tibial tuberosity, which is usually overlapped by the right leg in the original image. This also explains the slightly lower Dice coefficient for the subsequent reconstruction of the left side compared to the right side. However, this region is not of great importance for surgical planning purposes. Kasten et al. also reconstructed lower limb bones from biplanar radiographs and achieved a slightly higher Dice coefficient for the tibia [11]. However, they used single-leg radiographs and thus did not encounter the issue of sagittal superimposition.

Our framework was able to find clinically acceptable 3D surgical plans for all patients using 2D-3D reconstructed bone models. The achieved fitness values for both the ground truth and the reconstructed solutions differ only slightly, which demonstrates the usability of the reconstructed models for 3D preoperative planning. Furthermore, we calculated and reported the average differences of the correction angle as well as the positioning of osteotomy axis, osteotomy plane and fixation plate between the ground-truth and the reconstructed solutions. Some of these differences appear significant, but it should be kept in mind that the underlying problem is multi-objective and several solutions along the Pareto front can be considered as optimal.

While MA measurements were accurate for the reconstructed models through landmark localization, the differences for TS were significantly larger (mean 4.33°) and outside the acceptable range. This difference is entirely attributable to a correspondingly large difference in the plane that was fitted to the articular surface of the tibia, while the landmark localization (KJC, AJC) and thus the mechanical axis measurement were highly accurate. It is known that the plateau is not a flat plane but has medial and lateral variations in slope [20], which are difficult to discern in the sagittal projection [14]. Consequently, accurate 3D reconstruction of the articular surface from frontal and sagittal projections only is not possible. Additionally, in OA patients, the plateaus are often not even. The differences in measured TS also indirectly affect the orientation of the normal vector of the planned osteotomy plane, resulting in higher differences in the sagittal projection. Besides complex approached based on deep learning, a simpler approach could involve the detection a plane in the biplanar 2D image set, and subsequently use it for initialization and articular surface point selection in 3D.

While we designed a custom DRR protocol that respected the EOS imaging geometry, our study is limited by the use of DRRs, whose appearance differ from real EOS images. There are various approaches in the literature which will be leveraged to address this issue in future work. Kasten et al. trained a CycleGAN-based network for domain adaptation [11]. Another group proposed DeepDRR, a framework for deep-learning-based fast and realistic simulation of fluoroscopy and digital radiography from CT scans. Networks trained on DeepDRRs generalized well to real data without re-training or domain adaptation [18, 19]. Evaluating the performance of our framework using real EOS radiographs is part of our future work.

Furthermore, we would like to explore if PSIs created based on 2D–3D reconstructed bone models can be used as a surgical navigation technique. PSIs are frequently used navigation aids to ensure an accurate surgical execution of the preoperative plan. Since PSIs are molded to a patient's bone anatomy, they are directly dependent on the accuracy of the bone model reconstructed from the patient’s image data.

Finally, the standing position of the patients during image acquisition is not standardized. It is influenced by the instructions of the radiologist, the physical abilities of the patient and the magnitude of pain in the knee. Investigating the patient's position in the scanner would be very interesting in the future.

In summary, this study has demonstrated that the reconstruction of 3D bone models from biplanar radiographs is sufficiently accurate to be used in the context of 3D preoperative planning of HTO. The precise reconstruction of the TS remains challenging and needs to be addressed in the future.
